# Supplementary material for: Are carbon emissions trading and green financial instruments synergistic? -Comprehensive quantitative research based on content analysis
Source: PLoS One. 2024 Mar 7;19(3):e0298601. doi: 10.1371/journal.pone.0298601 (PMC10919601; doi:10.1371/journal.pone.0298601)
Supplement: S1 Data — (ZIP) [file pone.0298601.s001.zip › Data/地级市Z_F工作报告.zip!/╜¡╬≈/╕╙╓▌╩╨/└┤╫╘╣1⁄2╓┌║┼í╛┬φ┐╦╩2╛▌═°í┐_2011.docx]

各位代表：

　　现在，我代表市人民政府向大会报告工作，请予审议，并请市政协各位委员和列席会议的同志提出意见。

　　一、过去五年的工作回顾

　　过去的五年，在省委、省政府和市委的正确领导下，市政府带领和依靠全市人民，以科学发展观为统领，战胜雨雪冰冻和特大洪涝灾害，有效应对国际金融危机，全面完成“十一五”规划各项任务，主要取得了六个方面的成就。

　　（一）综合实力明显增强。全市生产总值2010年突破千亿元，财政总收入2008年突破百亿元，金融机构存款余额2009年底突破千亿元，成为全省第二个生产总值过千亿元、财政总收入过百亿元的设区市。预计五年固定资产投资总额可达到3000亿元。到年底，生产总值过百亿元、财政总收入过十亿元的县（市、区）将分别达到2个。

　　（二）经济结构进一步优化。三次产业结构调整优化为18.9∶44.4∶36.7，二产比重提高5.5个百分点。预计到年底，规模以上工业增加值、主营业务收入、利税总额分别比2006年增长2.5、3.4和2.9倍。稀土、钨两大产业集群主营业务收入年底有望超过500亿元，分别占全国的1/3，利税有望过100亿元。章源钨业成功上市。赣州成为世界最主要的脐橙主产区。果业、生猪等六大农业主导产业快速发展。贷款增量和增幅位居全省第二、周边市第一。

　　（三）城乡面貌显著变化。中心城区五年累计完成市政基础设施投资266亿元，建成区面积达到81.2平方公里，人口达到74.3万人。“治脏、治乱、治堵”工作取得实效，成功创建国家园林城市。全市城镇化率年底预计达44%。黄金机场建成运营，今年进出港人数有望突破50万人次，成为全省第二、周边市最大的机场；赣韶铁路建设和京九、赣龙铁路改造加快推进；瑞赣、石吉、鹰瑞、康大等高速公路建成通车，全市建成、在建高速公路总里程达到1070公里，约占全省的30%。建设农村公路16161公里，全面完成126座渡改桥任务，100%行政村实现道路硬化，立体化交通网络架构初步形成。社会主义新农村建设成效显著，整治建设村庄1万多个，村庄环境整治项目获“中国人居环境范例奖”。水利基础设施大为改观。全市森林覆盖率达到76.2%。主要污染物总量减排成效明显，实现省政府下达的“十一五”节能减排目标。主要河流断面水质达标率90%。环境空气质量保持优良。

　　（四）改革开放深入推进。政府机构、国有企业以及财税体制、医药卫生体制、投融资体制、林权制度改革扎实推进。开放型经济、非公有制经济加快发展。五年累计引进外资企业983家，实际利用外资39.69亿美元，引进投资5000万元以上内资项目630个。外贸出口总额增长3.7倍。成功申报建立国家级出口加工区，赣州开发区升格为国家级经济技术开发区。

　　（五）民生事业全面进步。2010年全市城镇居民人均可支配收入达到14203元，农民人均纯收入达到4182元，居民储蓄存款余额达到907亿元。财政民生支出比重逐年提高，主要民生指标全面完成。统筹城乡就业工作被列为国家试点。小额担保贷款发放总量连续五年排名全国地级市首位。保障性住房体系建设走在全国前列。在全省率先启动城乡社会养老保险制度全覆盖。为80周岁以上老人发放生活补贴。

　　各项社会事业全面发展。科技创新“六个一”工程扎实推进。各类教育持续健康发展。成功申报第一批国家公共文化服务体系示范区。现代赣南采茶戏《快乐标兵》荣获中宣部“五个一工程”奖。医疗卫生服务机构进一步完善。全民健身活动蓬勃开展。再创国家“双拥”模范城。顺利完成“五五”普法任务。人口计生、新闻出版、外事侨务、民族宗教、台湾事务、扶贫开发、水文气象、防震减灾、统计审计、检验检疫、质量监督、食品药品监管、民兵预备役、国防动员、人民防空、方志档案、妇女儿童、供销、物价、老龄、慈善、残疾人、红十字等工作取得新进步。连续四届荣获“全国社会治安综合治理优秀市”称号，连续两届荣获全国综治“长安杯”奖。

　　（六）政府自身建设得到加强。自觉接受人大法律监督和政协民主监督，认真听取各民主党派、人民团体和各界人士意见。人大代表建议、政协提案办复率100%，满意和基本满意率99.92%。建立了市政府常务会学法、重大行政决策合法性审查、重大政策文件审查等制度。市县两级行政服务中心功能进一步完善。应急管理体制进一步健全。政府信息和政务公开深入推进。反腐倡廉、惩治和预防腐败体系建设取得新成效。

　　各位代表，五年来的发展成就令人振奋。这些成绩的取得，是省委、省政府和市委正确领导的结果，是勤劳智慧的赣州人民同心同德、顽强拼搏的结果。在此，我谨代表市人民政府，向全市人民，向各民主党派、各人民团体和各界人士，向历任老领导、老同志，向驻市部队、武警官兵和公安司法干警，向为赣州发展作出贡献的企业家、科技工作者、进城务工人员，表示衷心感谢并致以崇高敬意！

　　五年来的发展历程令人难忘，我们积累了宝贵的经验和启示：必须牢固树立发展为先理念，始终坚持加快发展、转型发展不动摇，聚精会神搞建设，一心一意谋发展，在发展中促转型，在转型中谋发展；必须牢固树立生态为重理念，始终坚持既要金山银山、更要绿水青山不动摇， 走低碳绿色发展之路，推动经济与生态协调发展、人与自然和谐相处；必须牢固树立创新为魂理念，始终坚持改革开放不动摇，以改革破解难题、以创新激发活力、以开放汇聚能量，不断为发展注入不竭动力和强大活力；必须牢固树立民生为本理念，始终坚持改善民生、为民造福不动摇，着力解决人民群众最关心、最直接、最现实的问题，使发展成果最大限度地惠及人民群众。

　　我们也清醒地看到，经济社会发展中还存在不少困难和问题。一是经济总量偏小、人均水平较低，发展不快、发展不充分；二是经济结构、产业结构、税源结构不优，产业规模不大、集聚度不高、创新能力不强，重大项目和优势骨干企业较少；三是城镇化水平偏低，城市功能配套不完善，辐射带动力不强；四是部分群众生活比较困难，城乡居民收入水平与全国、全省差距较大；五是少数干部群众安于现状，自我发展的内生动力不强；六是政府服务水平和办事效率有待进一步提高。对此，市政府将采取有效措施，着力加以解决。

　　二、未来五年的工作任务

　　今后五年，是赣州坚持加快发展、推进转型发展的关键五年。我们既要看到近年来全市经济加速发展、基础设施更加完善、各方面优势蓄势待发，为新一轮大发展奠定了基础、创造了条件；又要看到宏观经济形势总体向好、产业转移加速推进、国家对革命老区和中部地区支持力度加大，为赣州发展带来难得机遇；还要看到资源环境要素制约趋紧、社会矛盾集中凸显、区域竞争更趋激烈带来的困难和挑战；尤其要看到人民群众对过上更加美好生活有着热切期待，对新一届政府推动加快发展、转型发展有着殷切期盼。我们务必切实增强加快发展的危机感、责任感和紧迫感，紧紧抓住难得的发展机遇期，以时不我待的精神全力以赴加快发展；努力在总量扩张中优化结构、提高效益，在坚持加快发展中推进转型发展，实现发展规模、发展层次和发展水平的新跨越；坚持把经济发展与改善民生有机统一起来，千方百计惠民生、保稳定、促和谐。

　　今后五年，全市经济社会发展的总体要求是：高举中国特色社会主义伟大旗帜，以邓小平理论和“三个代表”重要思想为指导，深入贯彻落实科学发展观，以科学发展为主题，以转变经济发展方式为主线，按照市第四次党代会部署，坚持加快发展，推进转型发展，牢固树立发展为先、生态为重、创新为魂、民生为本理念，大力实施新型工业化、新型城镇化、农业农村现代化、发展生态化战略，为建设创业、宜居、平安、生态、幸福赣州而努力奋斗。

　　经济社会发展的奋斗目标是：主要经济指标总量和人均水平在全省排位前移，增速高于全国、全省平均水平。到2016年，生产总值、固定资产投资均突破3000亿元，财政总收入突破500亿元，社会消费品零售总额突破1000亿元，城镇居民人均可支配收入突破32000元，农民人均纯收入突破10000元。三次产业结构调整为9∶51∶40，二产、三产对经济发展的带动力明显增强。力争通过五年努力，推动人口地域大市优势加速转化为发展优势，资源优势加速转化为产业优势，区位优势加速转化为竞争优势，生态优势加速转化为经济优势，让赣州更加富有实力、充满活力、独具魅力，在国际国内更有影响力。

　　实现上述宏伟蓝图，我们将重点做好六个方面的工作：

　　（一）以产业集群建设为突破，加快推进新型工业化，为加快发展、转型发展构建强大支撑

　　坚持以工业化为核心，以项目建设为抓手，主攻工业，强攻工业，推动矿产资源产业高端化、战略性新兴产业规模化、承接产业品牌化、传统优势产业高新化，在做大总量、优化结构中提升工业主导地位，力争未来五年，全市规模以上工业增加值年均增长20%以上。

　　做大做强稀土和钨及其应用产业集群。抓住国家加强稀土钨等战略性资源管理和整治整合的有利机遇，瞄准全球产业结构加速调整、战略性新兴产业加快发展为稀土和钨产业带来的广阔市场，推动资源重点向产业后端深度加工的科技型、创新型企业配置，推动资金向前端研发和后端加工环节流动，推动产业由主要生产初级产品、中间产品向生产高附加值的终端应用产品升级。重点发展稀土磁性材料及永磁电机、发光材料及绿色光源、稀土陶瓷材料及应用产品、钨硬质合金及刀钻具，力争五年内形成4万吨磁性材料、6000吨荧光粉、1000万台永磁电机、1万吨钨硬质合金的年生产能力，主营业务收入超千亿元，打造世界知名的稀土产业基地和钨产业基地。加大探矿找矿力度，增加可开发资源储备。积极引进市外资源，加快建设稀土、钨交易中心和集散中心，强化与全国重要资源地、重点企业联合报价机制，争取国家在赣州设立稀土资源和实物储备点，提高定价话语权和影响力。加快建设稀土、钨工程研究院等一批国家级、省级工程技术研究中心和实验室。采用市场配置、资产重组、入股参股等多种方式，整合现有采矿、分离、冶炼等生产能力，充分发挥重点企业在产业发展中的引领作用，引进一批战略投资者，培育一批集开采、冶炼、深加工、科研、贸易于一体的企业集团，提高产业集中度。

　　做大做强新能源汽车及其配套产业集群。深入研究国家新能源汽车产业发展规划，争取列为国家和省新能源汽车产业基地。加强与行业领先的研发机构、制造企业合作，在汽车动力电池、驱动电机和整车控制系统等方面突破关键技术，建设新能源汽车驱动电机及专用永磁材料产业基地、动力电池生产基地。支持关联企业自主成立新能源汽车企业联盟，引进国内外知名的整车和零配件生产企业，高端低端两路并进，逐步形成完整产业链。力争五年内形成新能源汽车20万辆、动力电池1万兆瓦、电机3000兆瓦、电控系统40万套的年生产能力，实现主营业务收入超千亿元，建成新能源汽车及配套产业基地。

　　做大做强铜铝有色金属产业集群。用好用活国家大力支持循环经济发展政策，积极开拓国内国外两个资源市场，积极申报国家级废旧金属拆解基地，建设2个年拆解能力30万吨以上的废旧金属再生利用园区。建立废铜收购交易中心，建设铜铝钴有色金属交易平台，增强资源保障能力。引进和培育一批终端应用产品生产企业，发展相关配套产业，拉长产业链条，提高产品附加值。重点扶持江钨新型合金50万吨铜杆铜线深加工、逸豪实业2万吨铜箔、赣州铝业20万吨铝板材等项目建设，力争五年内产业主营业务收入超千亿元，建成国内重要的铜铝有色金属循环经济产业基地。

　　加快培育电子信息、现代轻纺、食品工业3个主营业务收入分别超500亿元的产业集群，以及氟盐化工、生物制药、新型建材3个主营业务收入分别超200亿元的产业集群。继续推动机械制造等其他传统优势产业转型升级、加快发展。认真研究、及时跟进国家和省战略性新兴产业发展规划，以战略性新兴产业的大培育、大发展，推进转型发展，形成未来赣州发展的强大增长极。

　　加快赣州开发区建设步伐。着力完善基础设施，强化产业配套功能，进一步提升开发区重大项目和产业集聚的承载能力；进一步提升入园企业门槛，增加投资强度；进一步提升入园企业开工率、投产率和贡献率；进一步提升服务入园企业的能力和水平；推动中烟公司赣州卷烟厂等一批骨干企业加快建设，尽快投产，加速达产达标，重点培育稀土、钨、铜新材料，以及机械制造、食品、新能源汽车等支柱产业。力争到2016年主营业务收入过千亿元，税收过百亿元，成为赣州经济发展的重要支撑。

　　坚持信息化带动工业化，工业化促进信息化，推进工业化与信息化深度融合。重点围绕产业集群建设，推进设计研发、生产控制、市场营销及企业管理等方面信息化，加快工业园区数字化建设。用信息技术提升电子商务、现代物流、新兴业态、金融服务发展水平，推动经济社会各领域信息化。

　　（二）以特大城市建设为龙头，加快推进新型城镇化，为加快发展、转型发展铸造强力引擎

　　实施中心城区城市规模、城市人口倍增计划，做大做强做美中心城市，打造赣南城市群。

　　科学规划引领。强化“山、水、城、人”自然和谐相处理念，高起点完成特大城市总体规划及其专项规划。加强城市形态和功能设计，精心规划建设标志性建筑、主次干道、重点片区、特色街区和配套设施。注重差异性定位、功能性区分和个性化特色，打造高品质城市。优化“一核二廊三圈”①空间布局，着力推动特大型中心城市和瑞金、龙南都市区建设，支持信丰、兴国、于都、宁都等卫星城市和其他县城建设，促进赣县、南康、上犹与中心城区同城化发展，力争到2016年，中心城区建成面积达200平方公里、人口达200万的赣粤闽湘四省通衢的特大型、区域性、现代化中心城市，全市形成6个城区人口20万至50万、5个城区人口10万至20万的中小城市，30个重点小城镇，城镇化率提高到52%以上。

　　完善功能配套。强化城市基础支撑。完成特大型中心城市“四路三桥二互通”② 建设，形成各组团间快速通道网络。实施生态园林工程，保留自然山体，保护古树林木，开墙透绿透亮。推进峰山国家森林公园、蟠龙湿地公园建设，建成一批城市休闲公园、小游园、小广场。新建第二污水处理厂、第二垃圾处理厂。提升路网、水电、通信、燃气等配套能力，逐步实现公交、供水、供气同城同价。尽早启动城市备用水源点建设。推行“公交优先”，逐步建立公交政府补贴机制，优化线网结构，建成一批公交换乘枢纽站场和公共停车场，有效解决出行难、停车难问题。按照年吞吐量“远期500万人次，近中期200万人次”的规划目标，改扩建与特大城市相匹配的黄金机场。扩大支线航空试点城市效应，重点培育赣州至北京、上海、广深、南昌航线，努力突破直飞香港、台湾航线。推进火车站改造升级和新站建设，增开始发列车。加快赣龙铁路扩能改造、赣韶铁路、厦蓉高速赣崇段建设，尽快开工建设鹰瑞梅铁路，开工建设昌吉赣客专并争取延伸至深圳，力争到2016年铁路和高速公路通车里程分别突破800公里和1200公里，打造四省边际区域乃至我国东南地区的综合交通枢纽。加快华能瑞金电厂二期、西气东输、500千伏赣州东输变电工程、城乡电网改造等能源基础设施建设。加固城市堤防，中心城区防洪标准不低于50年一遇。

　　繁荣城市服务业。打造区域性金融中心、物流中心、旅游中心，新建改造一批农贸市场、新兴专业市场、综合市场和特色街区，构建与新型工业相配套、城市发展相协调、群众生活相适应的现代服务业体系。规划建设金融商务核心区，积极引进各类金融机构，发展新兴金融业态，建立金融资产、稀贵金属等金融交易市场。加快综合物流园区和六大物流节点③建设，形成以赣州为中心、辐射周边四省八市的“三小时物流服务圈”。科学整合红色、客家、宋城、生态等特色旅游资源，科学规划特色文化旅游产业园，科学打造精品线路；推进通天岩和瑞金红色景区创建5A级旅游区，建设著名旅游目的地。以完善的城市功能、优惠的政策，创造更多就业机会，留住进城农民工、外来就业创业人员、高校毕业生，集聚人气、商气、财气。

　　培育城市文化。传承历史文化，延续城市记忆。在城市扩容提质中彰显宋城文化、客家文化和水文化元素，建设“三江六岸”亲水公园、滨水走廊，打造“山、水、城”交相辉映、“人、园、景”相得益彰的品质城市。高位启动江南宋城核心区规划，加快实施保护性开发，整体打造成品味宋城古韵、品读客家风情、品尝赣南美食的文化产业园、旅游景区和影视基地。深入开发水文化资源。保护好福寿沟古城排水系统，发挥排水排涝、生态景观等综合效益，再续不涝之城历史。大力实施市民素质提升工程，弘扬客家精神，倡导城市文明，塑造市民新形象。

　　提升城市管理水平。深入推进“治脏、治乱、治堵”工作，打造卫生城市、秩序城市、畅通城市、宜居城市。加强城管队伍建设，加大城管综合执法力度，倡导文明执法理念，实施数字化、智能化管理，创建智慧城市④。规范城市文化管理。严厉查处违法用地、违法违规建设行为。争创国家生态园林城市、国家卫生城市和全国文明城市。

　　（三）以发展现代农业为抓手，推进农业农村现代化，为加快发展、转型发展打下坚实基础

　　坚持统筹城乡发展，突出发展现代农业，用工业的理念谋划农业，用工业园区的理念建设农业产业园，加快形成以城带乡、以工哺农、城乡互动、协调发展的城乡一体化新格局。

　　培育壮大以脐橙为主的农业主导产业。加强赣南脐橙产业的战略性研究，科学制定发展规划，加快产业现代化进程。加强标准化基地建设。调优品种结构，重点发展晚熟脐橙、杂交脐橙，扩大产业规模。加快中国（赣南）脐橙研究院、国家级脐橙工程技术研究中心建设，完善果技研究、推广、服务体系。大力发展加工、贮藏、物流等配套产业，建设赣南脐橙产业科技示范园。深度开发国内外市场，建立基本覆盖国内10万人以上城市和全球主要消费市场的营销体系。力争五年内脐橙产业产值翻番，成为品质世界最优、产业规模世界最大、产业综合竞争力世界最强、具有国际影响力和市场话语权的富民强市产业，让赣南脐橙成为赣州的亮丽名片，让橙色成为赣州的亮丽色调。培育壮大生猪、花卉苗木、蔬菜、油茶、工业原料林等主导产业，建设粤港澳优质商品猪、南国特色花卉苗木、精品蔬菜、高产油茶等生产加工基地。

　　建设高标准现代农业产业园。完善产业发展规划，制订产业建设标准，积极引导土地经营权向农业龙头企业、种养大户流转，积极引导农业龙头企业向园区集中，力争建设1至2个国家级现代农业高新产业园，支持有条件的县（市、区）建设一批现代农业示范园、科技园、生态园。坚持外销出口与本地供应并重，积极开展无公害农产品、绿色食品和有机食品生产基地建设，抓好中心城区商品蔬菜生产基地建设，努力把赣州打造成全国知名的“放心食品”生产示范区、优质供应区、消费安全区。大力引进一批产业关联度高、品牌知名度高、科技含量高的农产品生产企业，重点扶持一批有实力、有潜力、有前景的农业龙头企业。

　　加强以水利为重点的农村基础设施建设。贯彻落实《中共中央国务院关于加快水利改革发展的决定》，大规模开展农田水利建设，全面完成现有病险水库加固整治，加强中小河流治理，实现人水和谐。力争五年内解决228万农村居民和49.3万农村师生的饮水安全问题，新增和恢复灌溉面积44万亩，改善灌溉面积89万亩。每年完成10万亩标准农田建设。通过5至10年努力，基本建成“五大水利保障体系”⑤。加强农村乡际公路和通组公路建设，实现50%以上县道达三级公路标准，80%以上乡道达四级公路标准。组织实施好新一轮农村电网改造工程，基本解决农村电网建设水平低、供电不足、电压不稳的问题。

　　加快统筹城乡发展示范区建设。集中力量重点建设好储潭和三江等一批统筹城乡发展试验区，打造产业功能聚集、公共服务完善、基础设施健全、生态环境良好，集城市现代气息和乡村优美风光于一体的示范区。深化社会主义新农村建设，打造一批文化新村、产业新村、生态新村、旅游新村、文明新村。大力提升农民素质。完善市、县、乡、村四级培训网络，实施农业科技入户工程，加强农业实用技术培训。发展农村职业教育，完善县、乡、村公共就业服务机构和信息服务网络，加快农村富余劳动力转移，提高农民就业创业发展能力。加快发展农民专业合作经济组织，完善运行机制，提高农民参与市场竞争的组织化程度。

　　（四）以破解制约发展难题为关键，深入推进改革开放，为加快发展、转型发展提供强劲动力

　　坚持加快发展，推进转型发展，必须在改革上下真功夫，在开放上见新成效，在创新上有大举措。

　　深化各项改革。在继续推进行政管理体制、人事制度、医药卫生体制、财税体制等各项改革的同时，积极探索建立五大新机制，着力实施六大新举措，破解制约加快发展、转型发展的瓶颈问题。建立五大新机制。一是建立和完善政府部门服务企业发展、服务项目建设、服务县域经济和园区发展的长效机制，转变政府职能，下放管理权限，加强立项、审批、环评等关键环节的服务保障。二是建立鼓励和支持县域经济发展、争资争项、重点项目建设的激励机制，调动各方面的积极性竞相发展。三是建立与高校、科研院所合作创新机制，以项目为纽带，整合人才、设备等相关资源，聚集国内外知名专家学者围绕产业发展方向开展课题攻关，突破关键技术，带动产业发展。四是建立创新人才培养和引进机制，以及新型人事关系，加强人才队伍建设，大力引进与重点产业配套的各类专业人才，培养既懂科技又懂市场的创新创业人才，培养技术工人，为加快赣州发展提供人才支撑。五是建立存量土地流转机制，积极推进城乡土地增减挂钩、低丘缓坡综合开发利用试点。实施六大新举措。一是整合政府资源和国有资产、资本，拓宽融资渠道，组建国有控股投融资集团，发展产业投资、风险投资和股权投资基金，成立再担保公司，改制农信社组建市农村商业银行，做大地方银行，加快企业上市。加强政府性债务管理，切实做到举债有方、负债有度、还债有源、用债有效。二是研究具体措施加快区域性金融中心、物流中心、旅游中心建设，加速赣州现代服务业崛起。三是推进以户籍、产权制度为重点的城乡一体化综合配套改革，吸引农村劳动力向园区、城市转移，市外居民向赣州流动。四是扶持民营企业加快发展、返乡创业人员投资创业。五是鼓励国有企业做大做强，让国企焕发新活力，创造新业绩。六是细化赣州开发区帮扶生态环境优越县发展工业的具体举措。

　　扩大对外开放。坚持实施大开放主战略不动摇，坚持进一步解放思想、优化发展环境不停步，坚持把赣州打造成周边省市最具活力、最富商机、最低营商成本的开放高地不懈怠。紧靠省会城、面向 “长珠闽”、主攻港澳台，主动在鄱阳湖生态经济区建设中有更大作为。坚持引资与引智、引技联动，产业链招商、基地化招商、精细化招商并举，发挥各类商会、学会、协会、联谊会的积极作用招商，重点引进世界500强、央企、大型民企及配套企业，成建制承接产业转移。做大做强外贸进出口规模。未来五年，力争实现利用外资年均增长12%以上，外贸出口年均增长20%以上，新引进世界500强企业10家以上，国内500强、央企20家以上。

　　大力发展民营经济。进一步完善民营企业服务平台建设。出台民营投资导向目录，降低民间资本准入门槛，放宽市政公用设施的准入限制。积极拓宽创业融资渠道，着力破解民营企业融资难问题。加强民营企业家队伍建设，优化企业成长环境，支持本土企业、草根经济做大做强。鼓励在外创业的成功人士回乡发展，鼓励返乡人员投资创业。努力实现人员回转、资金回流、企业回迁，使赣州成为民营企业茁壮成长的沃土，成为百姓想创业、能创业、创成业的乐园。

　　（五）以做大总量为重点，培育壮大县域经济，为赣州加快发展、转型发展积蓄充足能量

　　坚持加快发展、推进转型发展，难点在县域，潜力也在县域。

　　找准路径引领县域经济发展。各地根据自身优势，找准产业定位，找准发展路径，探索符合自身发展的新路子。充分发现、挖掘和放大比较优势，发展特色经济，做大特色产业，走招商兴县、产业兴县、全民创业兴县之路，走差异化竞争、错位发展、竞相发展之路。支持中心城区周边县发挥区位优势，主动对接主导产业，着力发展现代服务业，加速融入中心城区经济圈。支持矿产资源优势县发展高端矿产产业，以资源引项目，以资源兴产业，推动资源优势加速转化为产业优势。支持生态环境优越县和文化资源丰富县，在保护好青山绿水、保护好历史文化的同时，发展生态文化旅游业，加速将生态优势转化为经济优势。支持人口大县向人力资源大县转变，以人力资源优势创造竞争优势，大力承接产业转移。支持传统产业优势县（市、区）做大做强机械制造、电子信息、现代轻纺、矿山机械、食品、家具等各具特色的传统产业，加快转型升级步伐。支持各地发展农业优势产业，使农业大县变成农业强县。支持各地大力开展职业技术培训和农民工实用技术培训，提高农村劳动力的就业能力和创业本领，加速农村富余劳动力转移。支持各地加快中小城镇建设，增强县域经济发展的承载力。支持各地依托工业园区加速新型工业化，力争五年内有2个园区主营业务收入分别超500亿元，另有5个园区分别超300亿元，形成一县一主业和若干区域特色经济板块。 推动全市县域经济主要指标三年翻番，力争到2016年，财政总收入县县过10亿元。

　　创新机制推动县域经济发展。建立科学完善、简便易行、导向明确的县域经济发展考核评价体系，强化财税、工业、项目建设等指标的考核，突出生态保护、优势资源开发利用等方面的考核，激励各县（市、区）以更大积极性，集中精力抓发展、集中力量抓发展、突出重点抓发展。建立县域经济发展工作定期调度机制，通过召开形势分析会、流动现场会等，及时通报情况，分析差距和问题，总结经验和做法。探索建立县（市、区）之间合作共建机制和利益共享机制，鼓励县与县之间打破行政界限联动发展，实现资源共享、信息互通、人才互用，合作争取重大政策，合作共建重大项目，合作开发优势资源。探索建立赣州开发区帮扶生态环境优越县的机制，鼓励生态优越县招大项目、招好项目、招产业配套项目落户赣州开发区，帮助生态环境优越县发展工业，做大经济总量，增加财政收入。

　　优化服务保障县域经济发展。从政策、资金、项目等方面加大对县域经济发展的扶持力度。积极探索强县扩权的有效途径，最大限度地扩大县（市、区）经济管理权限，增强县域经济自主发展能力。积极推动“中央苏区振兴规划”列入国家区域发展战略。加快建立市与县（市、区）之间易行有效的对接服务机制，采用市领导挂点帮扶、市直部门点对点服务、重大问题现场办公等方式，加强项目对接、服务对接和领导与企业的对接，积极帮助县（市、区）解决交通、电力、特色产业发展等方面的问题，共同努力争取实现“三通三有”，即县县通铁路，县县通高速公路，县县通管道天然气，县县有省级工业园区或特色产业基地，县县有成龙配套的电力电网，县县有一批投资10亿元以上、年纳税5000万元以上的大项目。通过县域经济强劲发力，推动赣州经济腾飞。

　　（六）以建设创业、宜居、平安、生态、幸福赣州为目标，着力改善民生，加快各项事业发展，让加快发展、转型发展成果惠及广大百姓

　　全心全意办好人民群众衣食住行、柴米油盐、安危冷暖等方面的事情，让生活在赣州的人民群众更有主人感、归宿感和幸福感。

　　实施八大工程，创造美好生活。力争到2016年，民生投入占财政支出比重提升到58%以上，五年累计突破1300亿元。一是实施“学有所教”工程。实施科教兴市战略，办好社会需要、人民满意、充满活力的教育，支持鼓励驻市高校做优做特做强。大力推进基本公共教育服务均等化。进一步加强教师队伍建设，解决农村偏远学校师资不足问题。建设城镇新区教育园区，满足农村进城学生就读需求。科学调整教育网点布局，合理配置优质教育资源，解决城镇学校“大班额”问题。推进义务教育学校标准化建设，全面消除农村学校D类危房⑥。每个乡镇建有1所以上达标公办幼儿园，每个行政村办好1所幼儿园，解决学前教育入园难、入园贵问题。二是实施“业有所就”工程。落实各项扶持创业就业的优惠政策，最大限度地激发全民创业就业热情。新建一批创业孵化基地、大学生和返乡农民工创业园、创业一条街和就业创业见习基地。加强职业教育园区建设，重点发展与产业集群建设相配套的职业教育。三是实施“劳有所得”工程。建立居民收入与经济增长挂钩、劳动所得与企业效益挂钩、工资增长与物价水平挂钩机制，实现城乡居民人均收入五年翻番。四是实施“住有所居”工程。加快建设廉租房、公租房、经济适用房，改造棚户区、城乡危旧房、红军烈士遗属及在乡老复员军人遗孀的土坯房。增加土地有效供应，坚决遏制土地闲置，制止捂盘惜售，有效抑制房价过快上涨，促进房地产市场健康发展。克服一切困难，按时保质保量完成年初下达的“三房一改”任务。五是实施“病有所医”工程。健全覆盖城乡的公共卫生服务体系、医疗服务体系、医疗保障体系、药品供应保障体系。完成市人民医院新院建设。每个县（市、区）至少有1所“二甲”以上综合医院，每个乡镇有1所卫生院，每个行政村有1所达标卫生室。六是实施“困有所济”工程。建立相对完善的社会救助体系，社会救助保障和抚恤补助标准逐年提高。实行医疗保险、工伤保险、失业保险、生育保险市级统筹。提高失地农民社会保障水平。建立低保与物价联动机制。完成1119个村整村推进扶贫开发任务，移民搬迁10万人。七是实施“民有所乐”工程。推进公共文化服务体系示范区建设。实现市有“四馆”（博物馆、图书馆、群艺馆、美术馆）、县有“三馆”（图书馆、博物馆、文化馆）、乡有“一站”（乡镇综合文体站）、村有“一室”（文化活动室），广播电视覆盖率显著提高；县（市、区）普遍建有“两场一馆一池”（体育场、健身广场、体育馆、游泳池），乡镇农民健身广场覆盖率达60%以上，村、社区体育设施覆盖率达50%以上。切实加强城乡户外公共文化体育设施建设，把文体设施建到群众家门口，丰富群众文化体育生活。八是实施“老有所养”工程。推行社会保障一卡通，实现城乡居民基本养老保险人员全覆盖，让社保阳光普照全民。为高龄老人发放生活补贴。确保五保户集中供养率达80%以上。积极探索城乡养老中心、养老公寓建设。整体推进农村敬老院（光荣院）和城乡社区服务中心建设，力争所有乡镇都有功能完备、设施齐全、环境优美的敬老院（光荣院），让农村鳏寡孤独老人安享幸福晚年。

　　推动各项事业协调发展，提高公共服务水平。完善创新体系，争创国家级创新型城市。启动赣南围屋申报世界文化遗产、七里窑遗址保护等工作。积极筹办好第十四届省运会，提高竞技体育水平。加强人口和计划生育工作，统筹解决人口发展问题，遏制出生人口性别比失衡，稳定低生育水平，提高出生人口素质。加快殡葬改革步伐，建设农村公益性墓地。抓好“菜篮子”工程建设，保障各类物资供应，控制物价过快上涨。实施妇女、儿童发展规划，关心和支持老龄、慈善与残疾人事业。发展好红十字博爱基金事业。继续做好国防动员、民兵预备役建设和“双拥”工作。深入开展民族团结进步创建活动，依法管理宗教事务，做好信教群众工作。进一步抓好外事侨务和台湾事务、防震减灾、统计、审计、人防、物价、地方志、档案等工作。

　　创新社会管理，维护安全稳定。加快推进城乡社区体制改革，完善社区服务功能，加强社区队伍建设，提高社区工作者和社会工作者待遇。推行社区网格化、智能化管理，提升服务和管理水平。加强民主法制建设，深入开展法制宣传教育。创新群众工作方式方法，完善矛盾纠纷排查预警、调解处置机制，建立健全人民调解、行政调解、司法调解工作机制，协调处理好各方面利益关系。建立安全生产长效机制，加强生产、消防、交通等领域的安全监管。抓好农产品安全监管，创建食品药品安全示范区，让老百姓吃上放心食品，用上放心药品。加强对流动人口、重点人群、复杂场所和虚拟社会的管理。完善重大事项风险评估和突发事件应急管理机制，提高突发事件监测预警、风险控制、应急救援和保障的水平。创新信访工作机制，提升信访工作科学化水平。完善治安防控体系建设，严厉打击各类违法犯罪活动，不断提高人民群众的安全感和满意度。

　　加强生态建设，创造秀美环境。保护和建设鄱阳湖生态经济区生态屏障。继续实施“一大四小”工程，五年完成绿化造林330万亩，森林覆盖率稳定在76%以上。加强“三江”源头、中小流域、饮用水源地等生态综合治理与保护，推动建立生态补偿机制。抓好废弃矿山生态环境恢复治理。加强地质灾害防治。培育发展生态农业、生态建筑业、生态旅游业、节能环保业，开发利用太阳能、水电、风电、生物质能等清洁能源。做好节能减排工作，推动政府机关为公共机构节能做表率。确保未来五年，全市主要河流断面水质达标率稳定在90%以上，环境空气质量优良率保持100%，主要污染物总量减排和节能减排达到国家和省控制目标，生态环境质量位居全省、全国前列，让赣州的山更绿、水更清、天更蓝。

　　三、大力弘扬苏区精神，再创新时期 “第一等的工作”，树立政府新形象

　　实现市第四次党代会描绘的宏伟蓝图，新一届政府必须在市委的正确领导下，在市人大法律监督和市政协民主监督下，大力弘扬宝贵而独特的苏区精神，加强对新政策、新知识、新理论成果的学习，加强对前瞻性、战略性、规律性问题的分析，加强对人民群众关注的热点难点问题的研究，全面提升加快发展、转型发展的能力，加强和创新社会管理的能力，做好新形势下群众工作的能力，和谐共事的能力，与新兴媒体坦诚沟通互动的能力，全力打造法治政府、责任政府、服务政府、效能政府、廉洁政府，再创新时期“第一等的工作”，树立和提升政府新形象。

　　（一）树立以人为本、亲民为民的政府新形象。“关心群众生活，注意工作方法”是毛泽东同志关于苏区群众工作的重要观点，也是新一届政府必须秉承的工作理念。不断强化群众观念。把人民群众放在心中最高位置，把为民造福当作最大的政绩，把群众满意不满意、高兴不高兴、答应不答应作为想问题、作决策的最高标准，真正做到权为民所用、情为民所系、利为民所谋。始终坚持群众路线。主动融入群众、亲近群众。不断完善市长热线、市长信箱办理机制，畅通民意表达渠道，让尊重人民群众、依靠人民群众成为工作的常态，最大限度地将人民群众的愿望集中起来、智慧凝聚起来、力量发挥出来。坚决维护群众利益。牢固树立“不与民争利”的理念，深化干部下基层 “送政策、送温暖、送服务”活动，实现干部联系服务群众全覆盖、常态化，坚持每年办好一批顺民意、解民忧、惠民生的好事实事，让广大群众共享改革发展成果。

　　（二）树立果敢坚毅、善抓落实的政府新形象。深入推进机关干部“治庸、治懒、治散”，切实转变政府机关干部作风。在改革进入攻坚期、矛盾处在凸显期的严峻考验面前，做到矛盾面前不回避，敢抓敢管、敢于碰硬；困难面前不退缩，敢闯敢试、敢为人先；责任面前不推诿，敢作敢为，敢于承担；在突发事件等急难险重问题面前，做到沉着应对，主动靠前。把所有心思用在加快发展、转型发展上，把所有智慧用在谋划项目、推动落实上。做到沉下身子抓落实。对市委决定的事不折不扣抓落实，政府集体研究的事分工合作抓落实，事关群众利益的事满腔热情抓落实，服务企业的事快办快结抓落实，认真做好每件事，认真干好每一天。减少文山会海，减少迎来送往，减少繁文缛节。少开会，开短会，开管用的会；少发文，发短文，发管用的文，切实把更多精力放在一线掌握情况上、一线解决问题上、一线干成事业上。提升效能抓落实。优化工作流程，创新服务方式，提高办事效率，努力打造服务“零缺陷”政府。适应网络发展的新趋势、媒体运用的新特点，加强电子政务建设，加强网上办事功能，推行网络问政，努力打造“24小时在线”政府。完善机制抓落实。建立群众诉求快速反应机制、企业发展帮扶机制、热点难题分级会诊机制、公众评议监督机制，真正做到服务发展不遗余力，服务企业给心给力，服务群众尽心尽力。

　　（三）树立和谐团结、清正廉洁的政府新形象。以宽容之心对待同志。倍加珍惜和维护班子团结，用坚强的党性保证团结，用高尚的人格增进团结，用共同的事业维护团结。做到高看组织、敬重同事、善待下属，学会欣赏别人的长处，学会分享别人的成功，切实提高政府一班人和谐共事的能力。以坦诚之心对待监督。自觉把监督视为事业顺利推进、个人健康成长的重要保证，认真办理人大代表议案、建议和政协提案，自觉接受法律监督、社会监督和舆论监督。依法加强政务公开和政府信息公开，让政府行政权力在阳光下运行。以敬畏之心对待权力。自觉筑牢思想防线、守住廉洁底线。坚持秉公用权、依法用权，切实履行“一岗双责”责任制，自觉遵守党风廉政建设各项制度规定，加强廉能风险防控，做清正廉洁的表率。

　　各位代表！

　　多少事，从来急；天地转，光阴迫；一万年太久，只争朝夕。让我们紧密团结在以胡锦涛同志为总书记的党中央周围，在省委、省政府和市委的正确领导下，始终保持勇立潮头的昂扬锐气、勇创一流的拼搏精神、勇攀高峰的壮志豪情，共同谱写赣州加快发展、转型发展的新篇章，为建设创业、宜居、平安、生态、幸福赣州而努力奋斗！
